# Supplementary material for: Octyl Gallate Induces Pancreatic Ductal Adenocarcinoma Cell Apoptosis and Suppresses Endothelial-Mesenchymal Transition-Promoted M2-Macrophages, HSP90α Secretion, and Tumor Growth
Source: Cells. 2019 Dec 30;9(1):91. doi: 10.3390/cells9010091 (PMC7016987; doi:10.3390/cells9010091)
Supplement: Supplementary file 1 [file cells-09-00091-s001.pdf]

Supplementary Materials:

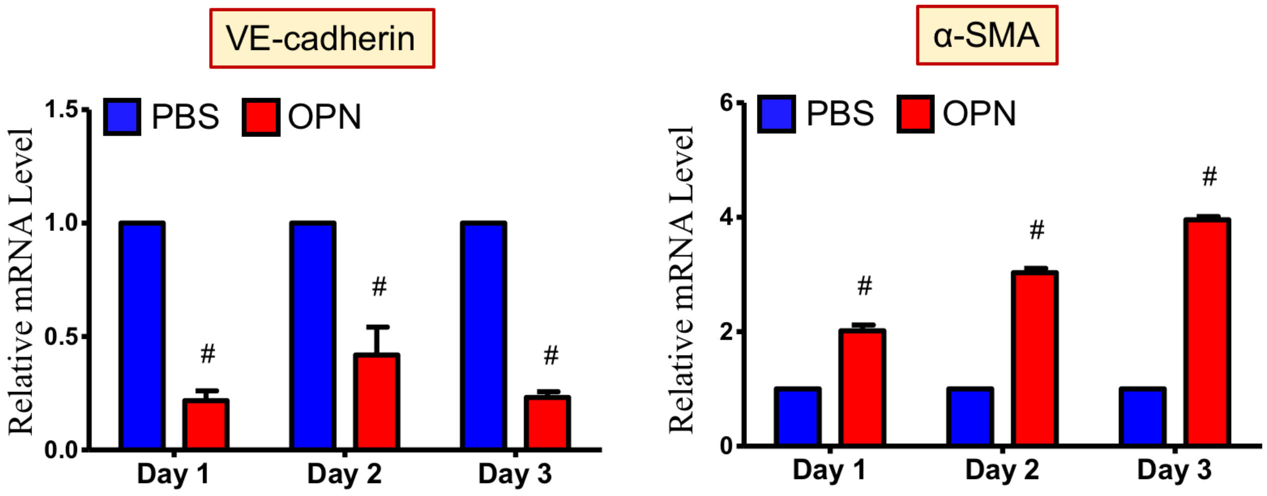

Supplementary Figure S1. EndoMT markers were induced and lasted for > 3 days in mouse endothelial cells. We have previously reported that OPN induced down-regulation of endothelial cell marker genes (VE-cadherin, Tie1, Tie2, and CD31) but up-regulation of mesenchymal cell marker genes (α-SMA and fibronectin) in mouse immortalized endothelial cell line 3B-11 [5]. In the present study, 3B-11 cells were seeded at a density of 2×10^6 cells per 10-cm dish and pre-incubated with 1% FBS-containing RPMI 1640 medium for 16 h. Cells were then added with 0.3 μg/mL of OPN for another 24 h. After washing twice with PBS, OPN-treated cells were incubated with fresh 1% FBS-containing RPMI 1640 medium for another 24, 48, or 72 h. The result revealed that OPN-induced down-regulation of VE-cadherin and up-regulation of α-SMA lasted for > 3 days.

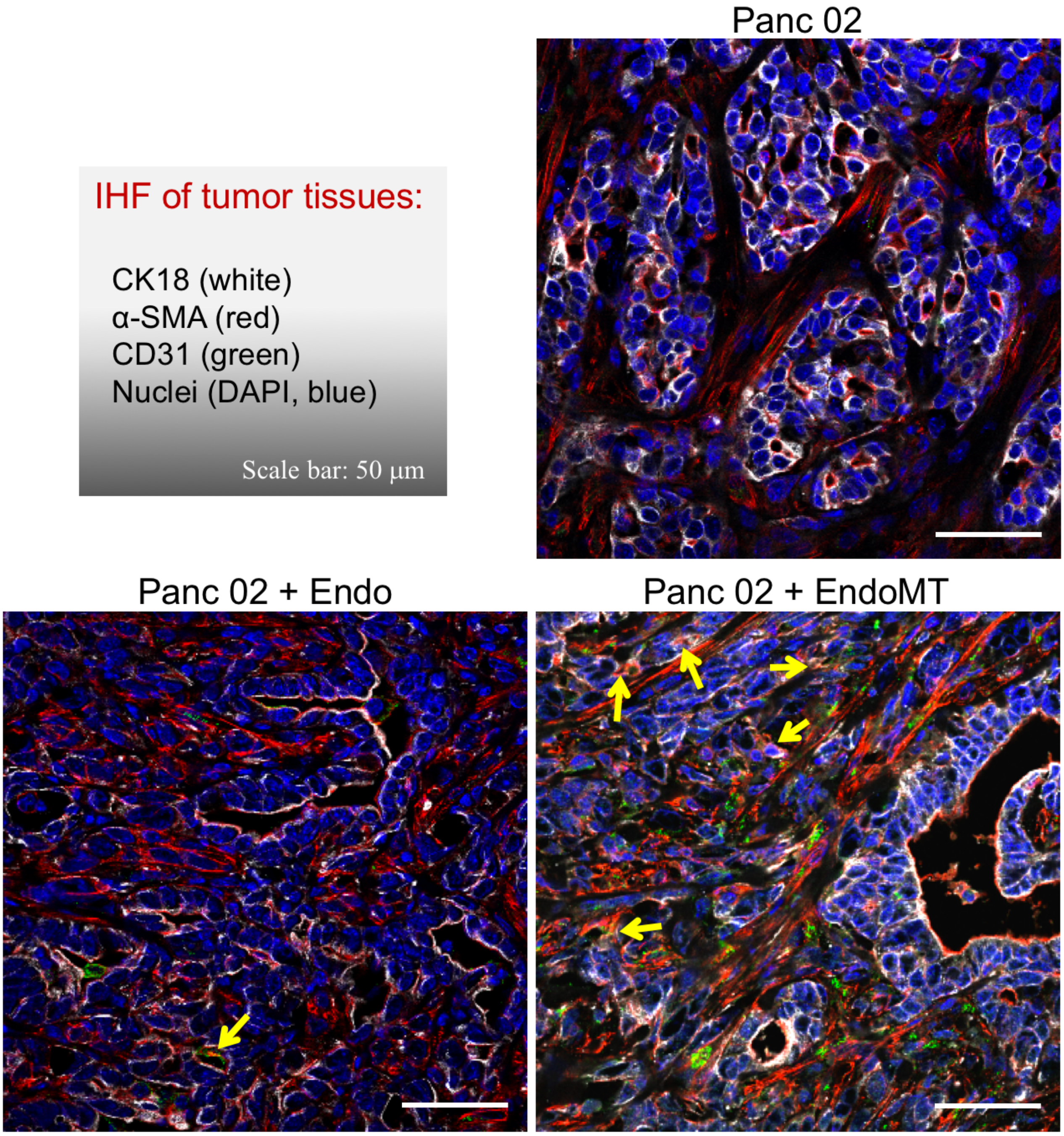

Supplementary Figure S2. Detection of EndoMT-derived cells in Panc 02 + EndoMT tumor model. Experimental mice were subcutaneously inoculated with Panc 02 (1×10^6 cells per mouse), Panc 02 (1×10^6 cells) plus PBS-treated 3B-11 (2.5×10^5 cells, denoted as Endo), or Panc 02 (1×10^6 cells per mouse) plus OPN-treated 3B-11 (2.5×10^5 cells per mouse, denoted as EndoMT). Mice were sacrificed and tumors were removed on Day 40 post-inoculation. The tumor masses in "Panc 02 + EndoMT" group comprised not only CK18-expressing Panc 02 cells and α-SMA$^+$ cells (infiltrating stromal cells or Panc 02 cells undergone EMT), but also α-SMA$^+$ and CD31$^+$ EndoMT-derived cells (indicated by yellow arrows), confirming the participation of EndoMT-derived cells in the tumors. Immunohistofluorescent staining was performed as described previously [5]. The primary antibodies included anti-CK18 (Abcam, #ab668; 1:100), anti-α-SMA (Abcam, #ab32575; 1:100), and anti-CD31 (Santa Cruz Biotechnology, sc-1505; 1:50) antibodies.

Supplementary Table S1. The primers and qPCR conditions adopted in this study.

Gene	Primer Sequence	PCR Condition & Product Size
GAPDH	Forward: 5'-GAA-GGT-GAA-GGT-CGG-AGT-3'<br>Reverse: 5'-GAA-GAT-GGT-GAT-GGG-ATT-TC-3'	95°C (30 sec), 56°C (40 sec), and 72°C (40 sec) for 40 cycles; 220 bp
α-SMA	Forward: 5'-TCC-AGA-GGC-ATA-GAG-AGA-CA-3'<br>Reverse: 5'-ACC-CTG-AAG-TAC-CCG-ATA-GA-3'	95°C (30 sec), 52°C (40 sec), and 72°C (40 sec) for 40 cycles; 222 bp
VE-cadherin	Forward: 5'-GTT-TCG-TGG-TGT-TAT-GTC-CT-3'<br>Reverse: 5'-AGT-TGT-TCC-GAG-TCA-CAA-AA-3'	95°C (30 sec), 49°C (40 sec), and 72°C (40 sec) for 40 cycles; 243 bp
TNF-α	Forward: 5'-CCC-AGG-CAG-TCA-GAT-CAT-CTT-3'<br>Reverse: 5'-TCT-CAG-CTC-CAC-GCC-ATT-3'	95°C (30 sec), 62°C (40 sec), and 72°C (40 sec) for 40 cycles; 140 bp
iNOS	Forward: 5'-GAG-AAA-GCC-CCC-TGT-GCC-3'<br>Reverse: 5'-TAC-CGC-TTC-CAC-CCT-GGC-3'	95°C (30 sec), 57°C (40 sec), and 72°C (40 sec) for 40 cycles; 422 bp
CD163	Forward: 5'-GAA-TAT-CAA-AAT-TGC-AAT-CAT-AGG-G-3'<br>Reverse: 5'-GTT-CAT-TTG-CTT-TGC-TTT-AGT-AAG-C-3'	95°C (30 sec), 62°C (40 sec), and 72°C (40 sec) for 40 cycles; 265 bp
TGF-β	Forward: 5'-CTA-CTA-CGC-CAA-GGA-GGT-CAC-3'<br>Reverse: 5'-TTG-CTG-AGG-TAT-CGC-CAG-GAA-3'	95°C (30 sec), 60°C (40 sec), and 72°C (40 sec) for 40 cycles; 249 bp
Arg1	Forward: 5'-ATG-CAG-AAC-TGT-GTG-GCA-TG-3'<br>Reverse: 5'-GGG-TTG-GTT-GAT-AAA-AGG-CA-3'	95°C (30 sec), 56°C (40 sec), and 72°C (40 sec) for 40 cycles; 254 bp
HSP90α	Forward: 5′-GCA-GAG-AAA-ACT-CTG-TCT-CG-3′<br>Reverse: 5′-CCT-GAG-AGA-ATT-CAC-TGT-GAG-C-3′	95°C (30 sec), 55°C (40 sec), and 72°C (40 sec) for 40 cycles; 163 bp
